# Descriptive analysis of service use covered by long-term care insurance in Japan - based on population-based claims data

**DOI:** 10.1186/1472-6963-14-S2-P125

**Published:** 2014-07-07

**Authors:** Nanako Tamiya, Masayo Kashiwagi, Hideto Takahashi, Haruko Noguchi

**Affiliations:** 1 Dept of Health Services Research, Faculty of Medicine, University of Tsukuba, Tsukuba, Ibaraki, Japan; 2 Association of Medical Science, Yokohama City University, Yokohama, Kanagawa, Japan; 3 Information Management and Statistics, School of Medicine, Fukushima Medical University, Fukushima, Japan; 4 Faculty of Political Science and Economics, Waseda University, Tokyo, Japan

## Background

Japan has the population with the highest proportion of aged people in the world and it rapidly continues to grow due to long life expectancy and a low birth rate, while traditional supports for elderly people are eroding. In response, the Japanese Government initiated mandatory public long-term care insurance (LTCI) in 2000. However, little has been published on the report of evaluation of LTCI with population-based data besides our previous report [1]. To make the provision of long-term care services effective, it is important for policy makers to have accurate evidence regarding the actual usage of services covered by public long-term care insurance (LTCI).

## Methods

The nation-wide claims data of February 2009, excluding data of some municipal bodies which were not available, was analyzed with official permission by Ministry of Health and Welfare. We evaluated the average expenditure and frequency of long-term care use covered by public LTCI and frequent patterns of services use by age, gender and care level.

## Results

In this study 620,091 males (34.2%) and 1,193,425 females (65.8%) were observed. The proportion of males decreased with age from 54% in the 65-69 age group to 16% for those 100 and older. The average expenditure on long-term care use per person is 10,540 yen for males and 11,055 yen for females. The expenditure increases with age for both genders, and males are more likely to use services than females under 75, which becomes reversed at 75 and older. However, the distribution of users’ age and gender varies by types of services. Regardless of age cohorts, males are more likely to use visiting nurse and visiting rehabilitation. The frequent patterns of service use are daycare only (15%), helper only (9%), daycare and rental device (7%), daycare and helper (6%) , rental device and helper (6%), but these patterns also vary by gender and age.

## Conclusions

This is the first study of detailed descriptions of service usage covered by the LTCI with population-based claims data. Policymakers and researchers can utilize these patterns of service use to predict future demands for long-term care and to conduct the policy evaluation.
